# Quality of Life in Caregivers of ADHD Children and Diabetes Patients

**DOI:** 10.3389/fpsyt.2016.00127

**Published:** 2016-07-25

**Authors:** Elisa Meirelles Andrade, Laysa Minella Geha, Paula Duran, Raphael Suwwan, Felipe Machado, Maria Conceição do Rosário

**Affiliations:** ^1^Child and Adolescent Psychiatry Unit (UPIA), Department of Psychiatry, Federal University of São Paulo (UNIFESP), São Paulo, Brazil

**Keywords:** quality of life, children, attention-deficit disorder and hyperactivity disorder, caregiver, diabetes

## Abstract

**Introduction:**

Studies have shown that the presence of attention-deficit hyperactivity disorder (ADHD) causes great impairment in academic, social, and professional activities as well as in the quality of life (QoL) of its patients. Similarly, the impact caused by other chronic disorders, such as diabetes, in the patient’s QoL has been emphasized in many studies. Despite its relevance, no study has yet investigated whether ADHD caregivers and diabetic patients would have similar QoL impairment.

**Objectives:**

This study was conducted in order to compare the QoL scores among ADHD caregivers and diabetic patients.

**Methods:**

We evaluated 63 caregivers of ADHD children treated at the Child and Adolescent Psychiatric Unit at the Federal University of São Paulo (UPIA-UNIFESP) and 52 adult diabetic patients. Subjects were assessed with the World Health Organization quality of Life-Bref Version (WHOQOL-BREF), the Beck and Hamilton depression scales, and the Adult Self-Report Scale.

**Results:**

When compared to the Brazilian normative data, ADHD caregivers had significantly lower scores in the social relations and environment WHOQOL domains. ADHD caregivers and diabetic patients had similar impairment in all WHOQOL domains except for the physical domain.

**Conclusion:**

ADHD affects the QoL of the patient’s caregiver, with similar impairment, when compared to the QoL of diabetic patients. These results emphasize the need for assessing QoL of the caregivers as part of the treatment strategies. They also emphasize the need for future studies with larger sample sizes comparing how the QOL is impacted in different chronic disorders.

## Introduction

Attention-deficit hyperactivity disorder (ADHD) is one of the most frequent mental disorders in the pediatric population, with prevalence rates ranging from 5 to 7.1% (1, 2). ADHD core features are inattention, hyperactivity, and impulsivity symptoms, causing significant impairment to the patient (3) and frequently persisting until adulthood. The presence of ADHD has a deep impact not only on the patients but also in their family members (4). For instance, having a child with ADHD increases family, marital, and parental problems, reduces parenting efficacy, and increases the level of parental stress (5). Parents of ADHD children have higher guilt, increased vulnerability to depression (6), greater alcohol consumption, and worse quality of life (QoL) (7, 8).

The concept of QoL has received increased attention over recent years. In general terms, QoL may be thought of as a multidimensional construct incorporating an individual’s subjective perception of physical, emotional, and social well being, including both a cognitive (satisfaction) and an emotional component (happiness) (9). Although QoL is influenced by many proximal (e.g., family and friendship) and distal (e.g., socioeconomic and cultural) factors, the presence of a chronic illness has been considered as one of the most potent risk factors for worse QoL scores (4, 9–14). Many authors have reported that chronic illnesses have an impact on many different aspects of the patient’s lives, which go beyond the core symptoms and impact their QoL (4, 9, 11–14). In studies using the World Health Organization Quality of Life-Bref Form (WHOQOL-Bref), one of the most used instruments to assess QoL, the scores are frequently lower in patients with chronic diseases, such as diabetes (9, 15–17).

Diabetes is a chronic disease considered as a serious public health problem. It is one of the most common chronic diseases worldwide, and its prevalence continues to increase mainly due to the changes in lifestyles resulting in physical inactivity and obesity. It was estimated that diabetes affected 285 million adults between 20 and 79 years in 2010 and will increase to 439 million adults by 2030 (18).

Diabetes affects a patient’s general health and well being in various ways, such as increasing the incidence of weight gain, functional disability, and mortality. Several studies have demonstrated that diabetes also causes a strong negative impact on the patient’s QoL (9, 15–20). Most studies found that being older, females, and having a low socioeconomic status were associated with worse QoL among diabetic patients (18, 21).

de Ornelas Maia et al. (21) found that diabetes was associated with depression and lower WHOQOL scores, especially in the physical, psychological, and social relationship domains. They also showed that the depressive and anxiety symptoms were closely associated with the lower QoL of diabetic patients. The biggest impairment was observed in the physical domain. Studies have suggested that improving patients’ health status and perceived ability to control their diabetes results in improved QoL. Therefore, it is important to use QoL measures when assessing patients with a chronic disease, such as diabetes. These measures can lead to different and better approaches to clinical care and social support (20).

Some studies have also demonstrated the association between the presence of chronic mental and physical disorders and QoL impairment (11, 14, 22–26). For instance, Akvardar et al. compared the QoL scores in three samples: (a) patients with psychiatric disorders, (b) diabetic patients, and (c) controls (24). They found that patients with mental illness reported less satisfaction in all WHOQOL domains compared with patients with diabetes and normal controls.

Similarly, some studies have also demonstrated that the QoL is also affected in caregivers of ADHD patients. Cussen et al. (27) evaluated the QoL, parental mental health, parenting styles, and parental relationship in 202 primary caregivers of ADHD children, compared with controls, and found that parents of ADHD children reported poorer family QoL in the domains of “emotional impact” and “impact on family activities,” less parental warmth, and higher parental depression and anxiety disorders. Kim et al. (14), in a naturalistic study with 75 parents of ADHD children, demonstrated that after 8 weeks of treatment with methylphenidate, children had a significant reduction in ADHD symptom severity, and that, their parents had improvements in depressive scores and on the physical, psychological, and environmental WHOQOL domains.

In summary, many studies have demonstrated that QoL may be impaired due to the presence of physical (15, 16, 20) and/or mental illness (13, 23, 24, 28) in the patient (11, 13, 23, 24, 29) and more specifically due to having ADHD (4, 22, 25–27). However, there are only five studies published in the literature investigating QoL in ADHD caregivers (11, 14, 30–32). Furthermore, none of these five studies compared the QoL scores between ADHD caregivers and patients with another chronic disease, such as diabetes.

Trying to address this question, this study was designed. The main hypotheses of the study were (1) both caregivers of ADHD children and diabetic patients will have significantly lower scores in all QoL domains, when compared to the general Brazilian population and (2) caregivers of ADHD children would have similar impairments in all QoL domains according to the WHOQOL-BREF when compared to diabetic patients. If proven true, the results would emphasize the need of assessing the QoL of ADHD caregivers, and the need for providing adequate treatment for them.

## Materials and Methods

This study consisted of a cross-sectional assessment of two samples. The first one comprised 63 adult caregivers of ADHD children diagnosed according to the DSM-IV criteria, who were being treated at the Child and Adolescent Psychiatry Unit (UPIA) at the Psychiatry Department of the Federal University of São Paulo (UNIFESP). The only inclusion criterion was to be a main caregiver or a child/adolescent with ADHD. The second sample was composed of 52 adults with Type 1 and/or Type 2 diabetes assessed in a reference center of cardiovascular diseases. The inclusion criteria were: having chronic diabetes (having the diagnosis for at least 4 years), not having any previous use of antidepressant drugs, not having a diagnosis of dementia, and not being treated for any psychiatric disorders. We also used the Brazilian normative data for comparisons between the two samples (33).

Data were collected after the approval of the Ethics Committee (IRB) of both sites according to the requirements of Resolution 196/96 of the National Health Council. After a detailed description of the study to all subjects, and the assurance that their decision to participate in the study would not interfere with their access to treatment, all subjects were asked to sign informed consent forms, authorizing their participation in the study.

### Instruments

#### World Health Organization Quality of Life-Bref

The shortened version, validated to the Brazilian population (33, 34), used to assess QoL in the caregivers, is an instrument composed of 26 questions, of which 2 are related to the individual overall perception about the QoL, and the remaining 24 are divided into four areas and represent the 24 facets that make up the original instrument (WHOQOL-100): domain I (physical) – focusing on pain and discomfort, energy and fatigue, sleep and rest, mobility, activities of daily living, dependence on medication or treatment, and work capacity; domain II (psychological) – emphasizing positive feelings, thinking, learning, memory and concentration, self-esteem, body image and appearance, negative feelings, spirituality, religion, and personal beliefs; domain III (social) – affairs, addressing personal relationships, support, social, and sexual activity; domain IV (environment) – assessing the physical safety and protection, home environment, financial resources, health and social care, opportunity to acquire new information and skills, participation in, and opportunities for recreation/leisure and physical environment. The instrument has construct validity with Brazilian samples. All items are answered according to Likert scales with scores ranging from 0 (worse) to 156 (best).

#### Adult Self-Report Scale

The Adult Self-Report Scale (ASRS) validated to the Brazilian population has 18 items that assess the presence and frequency of inattention, hyperactivity, and impulsivity symptoms according to the DSM-IV criterion A for ADHD diagnosis. It was used to assess ADHD symptoms in the caregivers of patients with ADHD. The scores range from 0 to 72 points (35).

#### Hamilton Depression Scale

The Hamilton depression scale (HAM-D) is a well-known scale used to assess the presence and severity of depressive symptoms. It has 17 items, each ranging from 0 up to 2 or 4. The total score can range from 0 (no symptoms) to 52 (very severe) (36).

#### Beck Depression Inventory

The Beck depression inventory (BDI) developed to assess presence and severity of depressive symptoms. This scale has excellent psychometric properties and has been validated for the Brazilian population. It contains 21 questions, each ranging from 0 to 3, with total scores ranging from 0 (no depressive symptoms) to 63 (very severe depressive symptoms) (37–39).

### Data Analysis

Socio demographic data were described as means, SD, and/or percentages, when applicable. Results provided by the WHOQOL-BREF were converted to scores, ranging from 0 to 100. Since the WHOQOL scores did not have a normal distribution, the non-parametric Mann–Witney test was used for the comparisons between each of the two case groups (ADHD caregivers and diabetes) and the Brazilian normative data. We compared each of the groups with the age-matched normative data according to the age ranges described by Cruz et al. (33).

The associations between depression, self-reported ADHD symptoms, and WHOQOL scores were analyzed using bivariate correlations (Pearson’s or Spearman’s when indicated). Since some authors suggest that depression generates impact in QoL (14, 15, 17, 40) scores, we also performed the analyses comparing patients with and without depressive symptoms. We used ANCOVA for the comparisons of the two case groups with each other (diabetes with and without depressive symptoms and ADHD caregivers with and without depressive symptoms). For these analyses, a score ≥16 in the BDI (38) and a HAM score ≥9 were used as cutoff points for depression.

A probability level of *p* < 0.05 was used to evince statistical significance. All statistical analyses were performed using SPSS v.16 software for Windows package.

## Results

Among the ADHD caregivers, there were 53 females (84.1%) and 10 males (15.9%). The diabetics group was composed of 34 females (65.4%) and 18 males (34.6%). There were no significant differences between the two groups in terms of gender, years of education, or socioeconomical status. There were significant differences among the groups regarding age (the diabetics group was older). The average age ± SD in the ADHD caregivers was 41.29 ± 10.18 years. In the diabetics, the mean age was 53.55 ± 10.61 years (Table 1). Because of this difference in age between the two groups and the relevance of this variable in QoL studies, age was used as a covariate in the correlation analyses and the comparisons between the ADHD and diabetes groups.

**Table 1:** **Sociodemographic characteristics of ADHD caregivers and diabetes patients**.

	ADHD caregivers (*n* = 63)	Diabetes (*n* = 52)	“*p*”
Age	41.29 (SD: 10.18)	53.55 (SD: 10.61)	<0.001
Female	84.1%	65.4%	
Schooling			
– Incomplete elementary school	20.4%	36.5%	0.174
– Completed elementary school	20.4%	23.1%	
– Completed high school	28.6%	26.9%	

World health organization quality of Life scores for each of the case groups were compared with the Brazilian normative data (Table 2). Comparing ADHD caregivers with the general population, the physical domain was higher in ADHD caregivers. In the social relations and environment domains, ADHD caregivers had lower scores than the general population. In the psychological domain, there were no significant differences between ADHD caregivers and the general population. Except for the physical domain (*p* = 0.09), diabetes patients had significantly lower scores in all other three domains when compared to the general population.

**Table 2:** **Comparison of mean WHOQOL-Bref domains between the general population and each of the case groups (ADHD caregivers and diabetes patients)**.

WHOQOL domains	Normative data mean (SD)	ADHD caregivers mean (SD)	Diabetes patients mean (SD)	“*p*”
Physical	(a) 57.9 (10.5)	70.3 (17.1)	–	0.0003
	(b) 59.2 (10.2)	–	54.8 (17.6)	NS
Psychological	(a) 62.5 (12.4)	64.1 (16.0)	–	NS
	(b) 63.0 (12.3)	–	60.2 (19.1)	0.03
Social relations	(a) 68.9 (21.7)	61.9 (21.3)	–	<0.001
	(b) 72.7 (17.3)	–	63.6 (18.9)	0.0002
Environment	(a) 59.7 (15.1)	55.8 (13.6)	–	0.02
	(b) 62.2 (15.9)	–	51.4 (18.4)	0.002

*(a) Normative data between the age range of 30–44 years old and (b) normative data between the age range of 45 and 64 years old*.

*NS = not significant*.

When comparing the four WHOQOL domains between ADHD caregivers and diabetes patients, the diabetics group had significantly lower scores in the physical domain (*p* < 0.01). There were no other significant differences between the two groups in the psychological relationships (*p* = 0.24), social relationships (*p* = 0.99), or environment domains (*p* = 0.16).

To investigate the effects of depressive symptoms on the QoL, we re-divided the two groups according to the presence of depressive symptoms higher than the cutoff points. It was observed that 55.8% of the ADHD caregivers and 44.2% of the diabetic subjects were above the cutoff points for depression (Table 3).

**Table 3:** **Comparison of WHOQOL-Bref domains mean scores of ADHD caregivers and diabetes patients with depressive symptoms**.

Domains	ADHD caregivers mean (SD)	Diabetes patients mean (SD)	Contrast	“*p*”
Physical	64.6 (17.7)	45.3 (13.6)	ADHD > diabetes*	<0.001
Psychological	57.3 (16.8)	50.8 (17.1)	ADHD = diabetes	NS
Social relations	54.5 (23.1)	59.7 (18.1)	ADHD = diabetes	NS
Environment	52.2 (13.4)	43.6 (12.1)	ADHD > diabetes*	0.02

*p < 0.05.

*Cutoff scores used for considering that a subject had depression: BDI score ≥16 and HAM score ≥9*.

*NS = not significant*.

As shown in Table 3, there were no significant differences between the two groups with depression in the psychological and social relationship WHOQOL domains. On the contrary, the physical and environmental WHOQOL domains were significantly worse in the depressed diabetic group (*p* < 0.001 and *p* = 0.02, respectively). When considering only the subjects without depression, only the physical WHOQOL domain was significantly different between ADHD caregivers and diabetes patients (*p* = 0.003). The other WHOQOL domains did not present significant differences between the non-depressed ADHD and Diabetes groups.

Significant negative correlations were found between self-reported symptoms of ADHD (according to the ASRS) in the ADHD caregivers group and the physical (*r* = −0.414, *p* = 0.01), psychological (*r* = −0.415, *p* = 0.01), and social relationships (*r* = −0.398, *p* = 0.001) and environment (*r* = −0.350 *p* = 0.01) WHOQOL-BREF domains.

## Discussion

This is the first study comparing the QoL scores between ADHD caregivers and the diabetic patients. Even though some studies have suggested that ADHD has an impact not only on the patient but also in the QoL of their caregivers, we wanted to investigate whether this impact would be similar to the one caused by a chronic disease, such as diabetes. As hypothesized, the results demonstrated similar impairments in the psychological and social relations WHOQOL domains, and therefore, emphasize how ADHD can affect the caregivers and the relevance of addressing this issue with them during treatment (14, 27).

When compared to the general population data, ADHD caregivers had even better scores than the general Brazilian population in the physical domain. On the contrary, diabetic patients had lower scores in the physical domain. It is possible to hypothesize that this difference might be secondary to the fact that ADHD caregivers were younger. This difference is expected since the onset of diabetes is more common after the age of 40 years (41), while ADHD caregivers seek treatment usually when their child is in elementary school (1). Some studies have shown that older patients with diabetes assessed their QoL as being significantly lower than younger patients (42, 43).

The higher impact of diabetes in the WHOQOL physical domain was probably secondary to the frequent limitations in their daily activities (42–44). de Souza (19) stated that physical capacity is one of the most impaired functions in diabetic patients, mainly caused by fatigue, weight loss, weakness, and despondency. These patients also report a greater negative impact on their QoL with particular reference to the effects of the disease on close personal relationships, sex life, self-confidence, motivation to achieve things, feelings about the future, freedom to eat, and freedom to drink (30).

Fleck et al. compared patients with clinical illnesses, psychiatric patients, and normal controls and found that the presence of a psychiatric disorder increased the risk for lower WHOQOL scores on the psychological relationship, social relationship, and environment domains (34). On the other hand, the normal controls had the best scores in the physical domain. In the current study, similar to psychiatric patients, ADHD caregivers also had lower WHOQOL scores in the social relationship and environment domains (when compared to the general Brazilian population), reinforcing the need for including the caregivers in the treatment strategies for ADHD patients.

Even though more recent studies have emphasized the impact of ADHD in the caregiver’s QoL, so far, there are only five studies published in the literature investigating QoL in ADHD caregivers (11, 14, 30–32). From these, only Xiang et al. (11) used the WHOQOL to evaluate 77 parents of ADHD children. Compared with the general Hong Kong population, they found significantly lower scores in all four WHOQOL domains. Furthermore, none of these five studies compared the QoL scores between ADHD caregivers and patients with a chronic disease.

In the current study, diabetic patients and ADHD caregivers presented similar impairment on the WHOQOL psychological and social relation domains. Even though this is the first study to compare the WHOQOL scores between these two groups, previous studies have reported that the presence of an ADHD child results in increased likelihood of disruptions in the family and the level of stress and burden in parents (42, 43). For instance, a recent study reported that 52% of parents of ADHD children suffered from a psychiatric disease (44), and that ADHD caregivers usually reduce contact with other people, interact only with their family members, and reduce social and labor activities. Chen et al. demonstrated that the four WHOQOL domains were significantly associated with depressive symptoms in mothers of ADHD children (30).

In the current study, the presence of depressive symptoms did not change the impact on the psychological and social relations WHOQOL domains. On the contrary, the physical and environmental WHOQOL domains were significantly worse in the depressed diabetic group.

We found significant negative correlations between the ADHD caregivers WHOQOL scores and the severity of ADHD symptoms in the caregivers. Some studies have reported that adults with ADHD usually fail to fulfill their responsibilities, resulting in academic, occupational underachievement, interpersonal problems, emotional difficulties, and other psychiatric comorbidities, such as depression, and most probably affecting their capacity to take care of a child with ADHD (45). Altogether, these results emphasize the need for assessing ADHD symptoms in the family members of ADHD children and including them in the treatment strategies, especially, when they also have ADHD symptoms or depression.

It is important to mention the limitations of the current study, such as the small sample sizes, what may limit the generalization of the results. Furthermore, the significant age differences between the two groups and the fact that we did not analyze the impact of the child’s ADHD symptom severity on the QoL of their caregivers are also worth mentioning. A recent study has reported that the child’s levels of attention and opposition symptoms had a significant interference in the psychological and environment domains of maternal QoL (30).

Despite these limitations, the current results reinforce the idea that ADHD impacts the whole family, and that being a caregiver of an ADHD child has an impact on their QoL similar to the one caused by having a chronic disease. They also emphasize the need for future studies with larger sample sizes comparing how the QoL is impacted in different chronic disorders. The results also support the relevance of involving all family members on the treatment strategies for ADHD treatment in order to improve results and promote positive outcomes. The present findings also suggest a need to improve the awareness of health professionals to identify high-risk ADHD caregivers for depression, ADHD and/or other disorders, and refer them as early as possible for treatment.

## Author Contributions

All authors participated in the conceptualization of the research, data collection, data analysis, paper writing, and paper critical review.

## Conflict of Interest Statement

The authors declare that the research was conducted in the absence of any commercial or financial relationships that could be construed as a potential conflict of interest.
